# Cellular senescence-related gene signatures in idiopathic pulmonary fibrosis: insights from bioinformatics

**DOI:** 10.3389/fimmu.2025.1557848

**Published:** 2025-06-10

**Authors:** Shuting Weng, Jingye Zuo, Jiali Mo, Leping Ye

**Affiliations:** Department of Pediatric Pulmonology, Children's Medical Center, Peking University First Hospital, Beijing, China

**Keywords:** idiopathic pulmonary fibrosis, cellular senescence, immune filtration, bioinformatics analysis, diagnostic markers, potential target

## Abstract

**Background:**

Idiopathic pulmonary fibrosis (IPF) is a progressive lung disease characterized by irreversible lung tissue scarring. Cellular senescence (CS) plays a significant role in IPF pathogenesis, yet the specific molecular mechanisms remain unclear. This study aimed to identify key CS-related differentially expressed genes (CS-DEGs) and investigate their potential as diagnostic biomarkers and therapeutic targets for IPF.

**Methods:**

Bioinformatics analysis was conducted on the GSE53845 dataset to identify CS-DEGs in IPF. Gene set enrichment analysis (GSEA), protein-protein interaction (PPI) network analysis, and functional enrichment analyses were performed to explore the biological functions and pathways associated with CS-DEGs. External validation of the identified CS-DEGs was performed using two independent datasets, GSE32537 and GSE24206. Immunofluorescence staining on lung tissue samples from IPF patients and normal controls was performed to validate the expression of key CS-DEGs.

**Results:**

A total of 122 DEGs were identified, and 8 core CS-DEGs were selected. CDKN2A, VEGFA, SOX2, and FOXO3 were validated as key CS-DEGs, with high diagnostic potential for IPF. Functional enrichment analysis revealed their involvement in critical biological pathways, including cellular senescence, immune response, and fibrosis regulation. Immunofluorescence staining confirmed higher expression of CDKN2A and SOX2, and lower expression of FOXO3 and VEGFA in IPF lung tissues compared to normal controls.

**Conclusion:**

This study highlights the significant role of CS-related genes in the pathogenesis of IPF and identifies four key CS-DEGs (CDKN2A, SOX2, FOXO3, and VEGFA) that could serve as potential biomarkers and therapeutic targets for IPF, providing a basis for further research.

## 1 Introduction

Idiopathic pulmonary fibrosis (IPF) is a chronic, progressive interstitial lung disease characterized by irreversible scarring of lung tissue, ultimately leading to respiratory failure (1–3). The prognosis is poor, with a median survival time of 2–3 years after diagnosis (2, 4). While the exact cause is unknown, age is a major risk factor (5, 6). A hallmark of IPF is the repeated injury to alveolar epithelial cells, which activates fibroblasts and promotes their differentiation into myofibroblasts. These myofibroblasts produce and deposit excessive extracellular matrix (ECM), resulting in progressive fibrosis (7–9). Despite advances in understanding these processes, the precise mechanisms underlying IPF remain unclear. Currently, lung transplantation is the only curative treatment, and antifibrotic agents such as pirfenidone and nintedanib can only slow disease progression without reversing fibrosis (2, 4, 5, 10). Therefore, identifying novel biomarkers and therapeutic targets is crucial to improve diagnosis and treatment outcomes in IPF.

Cellular senescence, a state of irreversible cell cycle arrest triggered by various stressors, plays a pivotal role in aging and age-related disease (11, 12). In IPF, accumulating evidence suggests that senescent cells contribute to disease progression by promoting inflammation, enhancing ECM deposition, and disrupting immune homeostasis (1, 12). These cells secrete a variety of pro-fibrotic factors collectively known as the senescence-associated secretory phenotype (SASP), which further drives fibrosis (1, 13). However, the specific cellular senescence-related genes (CSGs) involved in IPF and their potential diagnostic or therapeutic relevance remain largely unexplored.

In this study, we performed a comprehensive bioinformatics analysis to identify differentially expressed genes (DEGs) and cellular senescence-related DEGs (CS-DEGs) in IPF using publicly available transcriptomic datasets. We assessed their diagnostic value, investigated potential regulatory mechanisms involving transcription factors (TFs) and microRNAs (miRNAs), and identified potential therapeutic agents targeting key CS-DEGs. Finally, immunofluorescence staining was used to validate the expression of key CS-DEGs in lung tissue samples from IPF patients.

This research aims to provide new insights into the molecular mechanisms linking cellular senescence and IPF, while identifying potential biomarkers and therapeutic targets to facilitate early diagnosis and more effective treatments.

## 2 Materials and methods

### 2.1 Data collection and preprocessing

**Figure 1:**
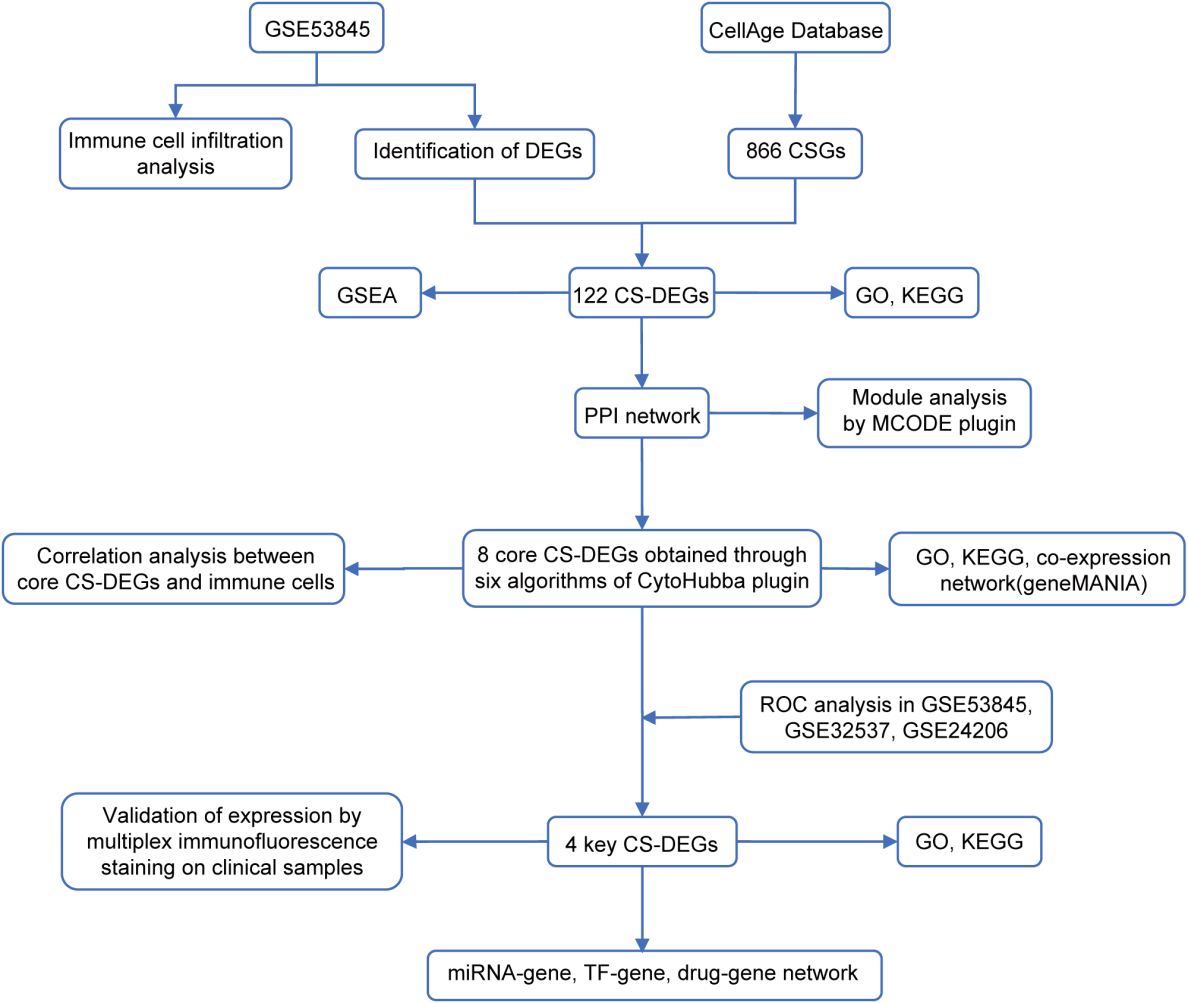
The workflow chart of this study.

Gene expression datasets GSE53845 (platform GPL6480), GSE32537 (platform GPL6244), and GSE24206 (platform GPL570) were obtained from the Gene Expression Omnibus (GEO) database (https://www.ncbi.nlm.nih.gov/geo/). GSE53845, used as the training set, includes transcriptomic data from lung tissue of 40 IPF patients and 8 healthy controls. GSE32537 and GSE24206 served as validation sets, containing 119 IPF patients and 50 healthy controls, and 17 IPF patients and 6 healthy controls, respectively. A set of 866 CSGs was retrieved from the CellAge database (14) (https://genomics.senescence.info/cells/). All datasets were normalized using the ‘normalizeBetweenArrays’ function from the ‘limma’ R package (version 3.62.2) and underwent quality control to ensure consistency and exclude outliers before analysis. The workflow chart of this study was shown in **Figure 1**.

### 2.2 Identification of DEGs and CS-DEGs

DEGs between IPF patients and healthy controls were identified using the ‘limma’ package in R software (version 4.4.0), with the selection criteria of |log2 FC| > 0.5 and adj.P.Val < 0.05. The intersection of DEGs with CSGs was then obtained using the ‘VennDiagram’ package to identify CS-DEGs.

### 2.3 Gene set enrichment analysis

GSEA was conducted to investigate biological processes associated with DEGs, using the ‘clusterProfiler’, ‘enrichplot’, and ‘ReactomePA’ packages in R. The analysis utilized the ‘h.all.v7.0.symbols.gmt’ as the reference gene set. In this approach, DEGs were ranked by their expression differences between IPF patients and healthy controls. Enrichment at the top of the ranked list indicated upregulated pathways, while enrichment at the bottom indicated downregulated pathways.

### 2.4 Functional enrichment analysis

To investigate potential functions and pathways associated with specific genes, Gene Ontology (GO) and Kyoto Encyclopedia of Genes and Genomes (KEGG) pathway enrichment analysis were conducted using the ‘clusterProfiler’ packages in R. GO analysis included three categories: Molecular Function (MF), Cellular Component (CC), and Biological Process (BP).

### 2.5 Protein-protein interaction network construction and module analysis

CS-DEGs were uploaded to the Search Tool for the Retrieval of Interacting Genes (STRING; http://string-db.org) database (15), with an interaction score threshold of 0.4 to construct the PPI network. The network was visualized using Cytoscape software (version 3.10.2) (16). The Molecular Complex Detection Technology (MCODE) plugin in Cytoscape (https://apps.cytoscape.org/apps/mcode) was applied to identify densely connected clusters within the PPI network, with parameters: K-core=2, degree cut-off=2, and max depth=100.

### 2.6 Identification and analysis of core CS-DEGs

Core CS-DEGs were identified using the CytoHubba plugin in Cytoscape (http://apps.cytoscape.org/apps/cytohubba), applying six algorithms (Betweenness, Closeness, Degree, MCC, Radiality, and Stress). The top ten genes from each algorithm were selected, and the intersection of these genes was used to identify the core CS-DEGs. A co-expression network of these core CS-DEGs was then constructed using the GeneMANIA (17) (http://www.genemania.org/) database to explore their interrelationships.

### 2.7 Immune cell infiltration analysis

Immune cell infiltration in each sample was analyzed using the CIBERSORT algorithm to estimate the proportions of 22 immune cell types. The results were visualized using the ‘ggplot2’, ‘ggpubr’, and ‘pheatmap’ R packages. Correlation analysis between 22 immune cell types and core CS-DEGs was performed and visualized using the ‘psych’ and ‘corrplot’ R packages.

### 2.8 Identification of key CS-DEGs and evaluation of their diagnostic value

To systematically evaluate the diagnostic potential of core CS-DEGs in IPF, a multi-stage analytical framework was implemented. First, receiver operating characteristic (ROC) curve analysis was performed on both training set (GSE53845) and validation sets (GSE32537 and GSE24206). Genes with an area under the ROC curve (AUC) > 0.7 across all datasets were identified as key CS-DEGs. Subsequently, the least absolute shrinkage and selection operator (LASSO)-penalized logistic regression (α = 1) was applied via the ‘glmnet’ R package to optimize multi-gene model, employing stratified 5-fold cross-validation to mitigate overfitting. The regularization parameter (λ) was selected from 10 logarithmically spaced values (0.001-0.1) through cross-validated AUC maximization. The multi-gene model was then rigorously validated in two validation sets. To estimate the confidence interval (CI) of the AUC, we applied Bootstrap resampling (1000 iterations) to compute the 95% CIs for all AUC estimates. Finally, statistical comparisons between multi-gene and single-gene models were conducted using DeLong’s test.

### 2.9 Construction of regulatory networks and exploration of potentially effective drugs for key CS-DEGs

TFs and miRNAs regulating key CS-DEGs were obtained from the TRRUST database (http://www.grnpedia.org/trrust) and miRTarBase (version 9.0) (https://mirtarbase.cuhk.edu.cn/), respectively. Potentially effective drugs targeting key CS-DEGs were identified through the Drug-gene interaction database (DGIdb)(https://dgidb.genome.wustl.edu/). The regulatory networks and drug-gene networks were visualized using Cytoscape software.

### 2.10 Multiplex immunofluorescence staining

Lung tissue samples used in this study were archived, formalin-fixed paraffin-embedded (FFPE) specimens obtained from the Department of Pathology at Peking University First Hospital. These included samples from three patients diagnosed with IPF and three adjacent normal lung tissues from age-matched lung cancer patients, with normal tissues collected at least 1 cm away from the tumor margin. All samples were originally collected between 2018 and 2024 as residual materials from routine clinical diagnosis and treatment, and were de-identified prior to analysis. Histological diagnoses of all samples were confirmed by hematoxylin and eosin (H&E) staining. This retrospective study was approved by the Ethics Committee of Peking University First Hospital (Approval No.: 2025-092-001). In accordance with institutional policy and local regulations, and given the retrospective nature and use of de-identified human tissue, the Ethics Committee granted a waiver of written informed consent. The study was conducted in accordance with the principles of the Declaration of Helsinki.

Multiplex immunofluorescence staining was performed to validate the expression of the four key CS-DEGs (CDKN2A, FOXO3, SOX2, and VEGFA) in IPF lung tissues. In short, formalin-fixed, paraffin-embedded lung tissue sections (4 µm thick) were deparaffinized, rehydrated, and subjected to antigen retrieval using EDTA (pH 8.0) by microwave treatment. After washing with PBS, sections were incubated overnight at 4°C with primary antibodies: anti-CDKN2A (Abcam, ab185620, 1:1000), anti-FOXO3 (Abcam, ab23683, 1:1000), anti-SOX2 (Thermo, PA1-094X, 1:1000), and anti-VEGFA (Affinity, AF5131, 1:2000). Following incubation with primary antibodies, tissue sections were washed with PBST and then incubated with HRP-conjugated secondary antibody for 30 minutes at room temperature. After additional washing, sections were incubated with different fluorescent dyes and counterstained with DAPI for 10 minutes. Immunofluorescence images were captured using a confocal microscope (AFBIO, AF-NE610-5-6). Expression levels of CDKN2A, FOXO3, SOX2, and VEGFA were quantified using ImageJ software and GraphPad Prism (version 10.1.2).

### 2.11 Statistical analysis

All statistical analyses were performed using R (version 4.4.1) (https://www.r-project.org/) and GraphPad Prism (version 10.1.2). Differences between two groups were assessed using the Wilcoxon rank-sum test or Student’s t-test, depending on the data distribution. The correlation between variables was analyzed using Pearson or Spearman correlation tests. All p-values were two-sided, with p < 0.05 considered statistically significant.

## 3 Results

### 3.1 DEGs identification and GSEA

**Figure 2:**
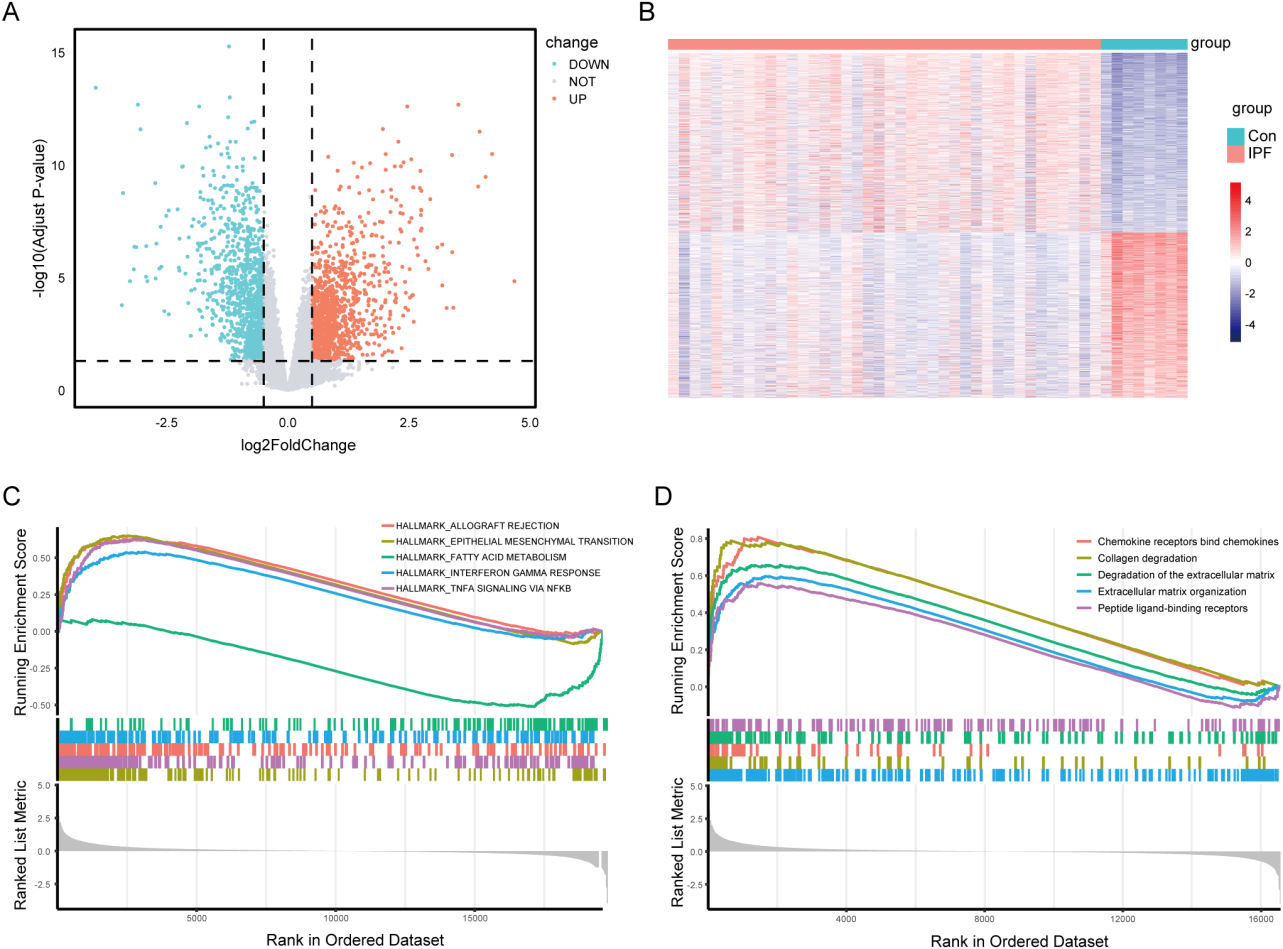
Identification of differentially expressed genes (DEGs) and Gene Set Enrichment Analysis (GSEA) in IPF. **(A)** Volcano plot showing the DEGs identified from the GSE53845 dataset, with |log2 FC| > 0.5 and adjusted p-value < 0.05 as the threshold. Red dots represent upregulated genes, while blue dots represent downregulated genes. **(B)** Heatmap illustrates the expression levels of the identified DEGs across the samples. Gene set enrichment analysis (GSEA) using reference gene sets from HALLMARK database **(C)** and REACTOME database **(D)** for DEGs in IPF.

After normalizing the microarray data, we performed a differential expression analysis using the GSE53845 dataset. A total of 2175 DEGs were identified, comprising 1133 up-regulated genes and 1042 down-regulated genes (**Figures 2A, B**). To further investigate the biological significance of these DEGs in the pathogenesis of IPF, we conducted GSEA using both the HALLMARK and REACTOME gene sets. The analysis based on the HALLMARK gene set revealed that the DEGs were significantly enriched in items related to allograft rejection, epithelial-mesenchymal transition (EMT), fatty acid metabolism, interferon-gamma response, and TNF-alpha signaling via NF-κB (**Figure 2C**). In addition, GSEA using the REACTOME gene sets indicated that the DEGs were mainly associated with chemokine receptors binding chemokines, collagen degradation, degradation of the extracellular matrix, and extracellular matrix organization (**Figure 2D**).

### 3.2 CS-DEGs identification and function enrichment analysis

**Figure 3:**
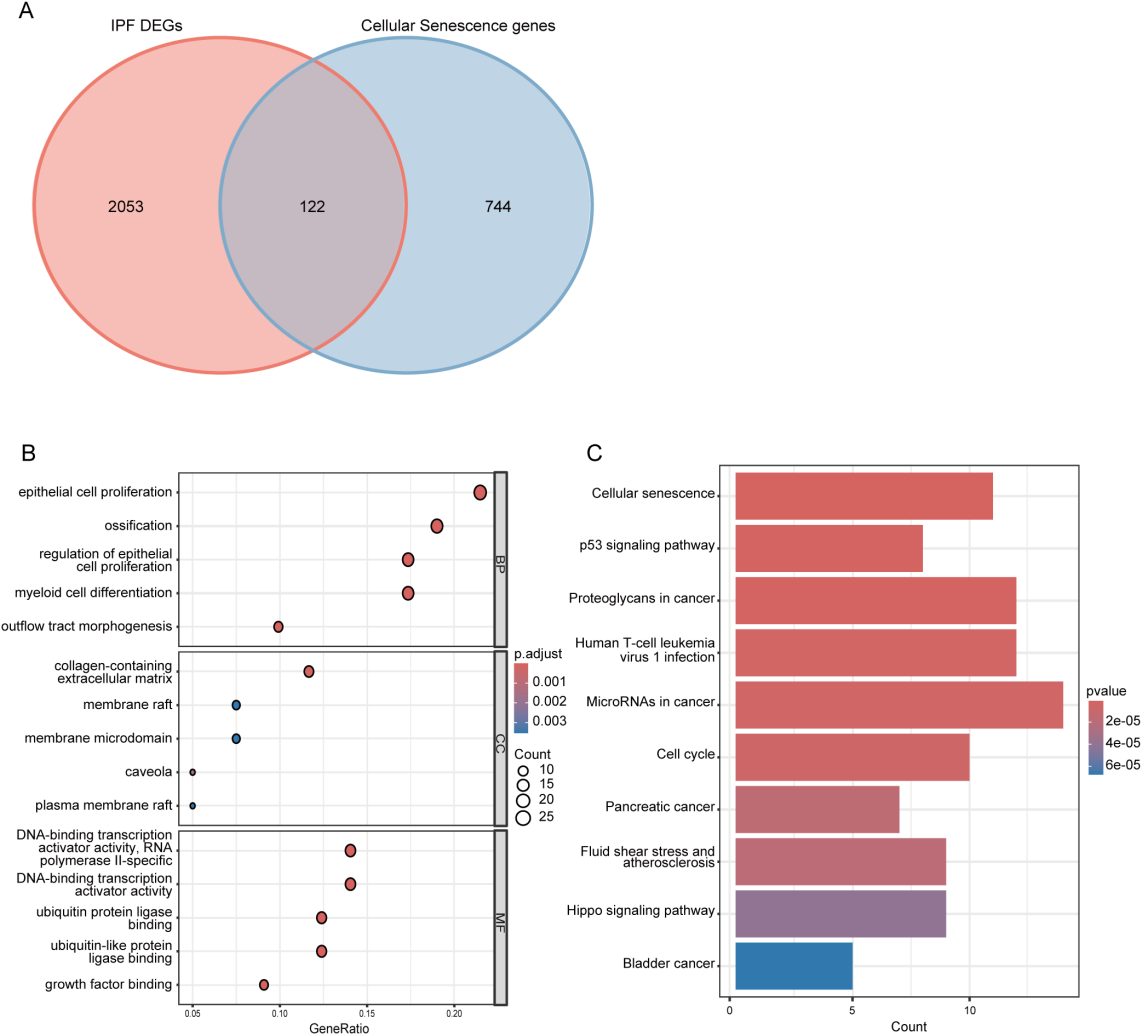
Identification and functional enrichment analysis of cellular senescence-related differentially expressed genes (CS-DEGs). **(A)** Venn diagram showing CS-DEGs. **(B)** Bubble plot presenting the Gene Ontology (GO) analysis of CS-DEGs. The size of the bubbles indicates the count of genes in each GO category, while the color of the bubbles represents the adjusted p-value. **(C)** Bar chart illustrating the results of KEGG pathway enrichment analysis for CS-DEGs. The length of the bars corresponds to the number of genes in each pathway, and the color of the bars indicates the p-value for each pathway. BP, Biological Process; MF, molecular function; CC, cellular component.

By intersecting 866 CSGs with the 2175 DEGs identified in the first part of our analysis, we obtained 122 CS-DEGs (**Figure 3A**). To gain insights into the biological functions and pathways involving the CS-DEGs, we performed GO and KEGG pathway enrichment analysis. The GO analysis revealed that, in the BP category, the top 5 enriched terms were epithelial cell proliferation, ossification, outflow tract morphogenesis, regulation of epithelial cell proliferation, and myeloid cell differentiation. In the CC category, genes were enriched in collagen-containing extracellular matrix, caveola, membrane raft, membrane microdomain, and plasma membrane raft. For the MF category, the top 5 enrichment terms included growth factor binding, ubiquitin protein ligase binding, ubiquitin-like protein ligase binding, DNA-binding transcription activator activity (RNA polymerase II-specific), and DNA-binding transcription activator activity (**Figure 3B**). KEGG pathway analysis showed that the CS-DEGs were mainly enriched in cellular senescence, p53 signaling pathway, proteoglycans in cancer, human T-cell leukemia virus 1 infection, and microRNAs in cancer (**Figure 3C**).

### 3.3 PPI network analysis

**Figure 4:**
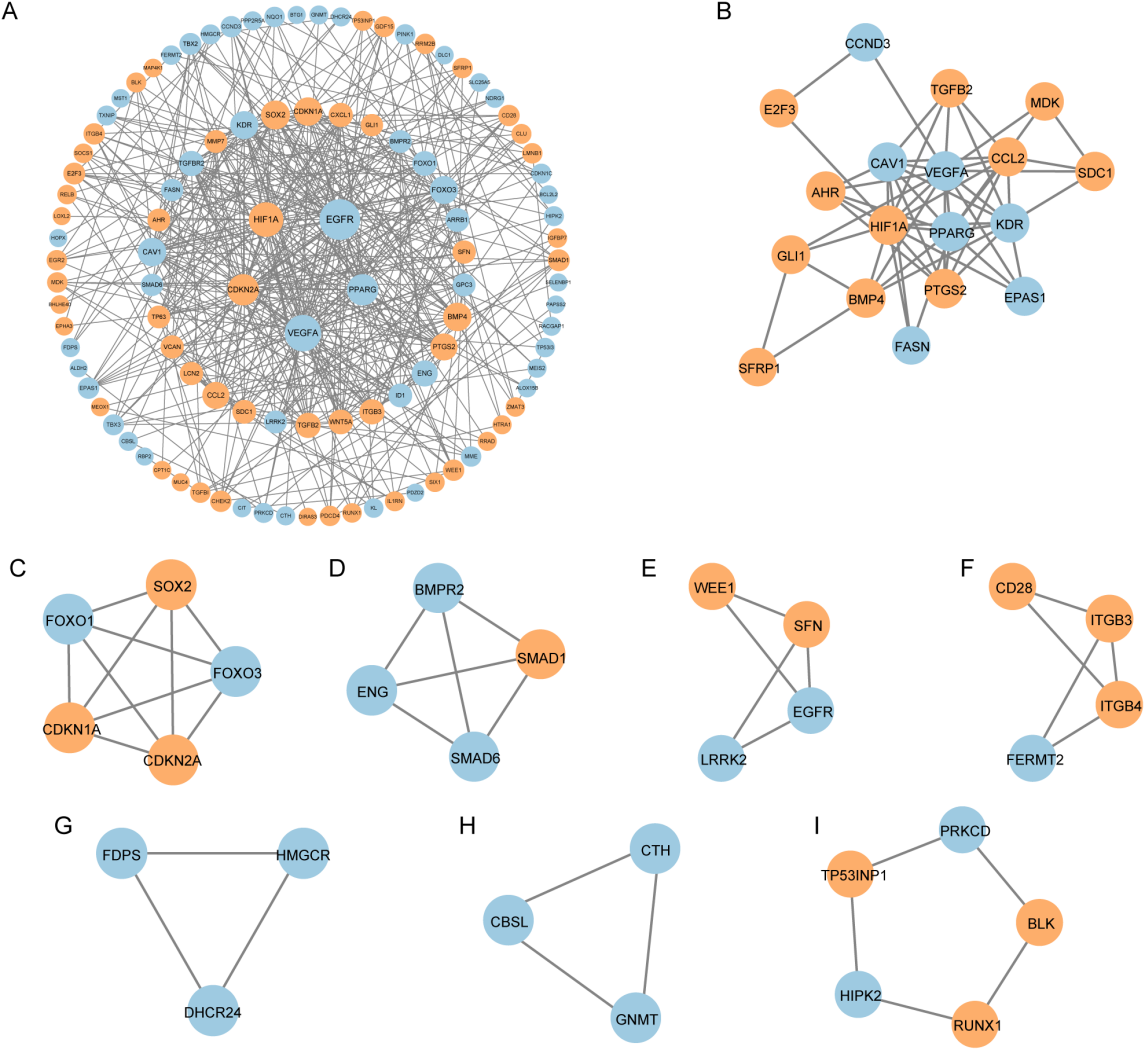
Protein–protein interaction (PPI) analysis of CS-DEGs. **(A)** PPI network of cellular senescence-related differentially expressed genes (CS-DEGs). Nodes represent proteins, and edges represent interactions between proteins. **(B-I)** Clusters 1–8 identified by MCODE plugin from the PPI network. The clusters are arranged in descending order of their scores, with each cluster representing a tightly interconnected group of genes.

We constructed a PPI network for the 122 CS-DEGs using the STRING database, resulting in a network of 106 nodes and 468 edges (**Figure 4A**). Using the MCODE plugin in Cytoscape, we identified 8 tightly interconnected gene modules, comprising 46 nodes and 53 edges (**Figures 4B–I**), which may represent key functional clusters relevant to cellular senescence in IPF.

**Figure 5:**
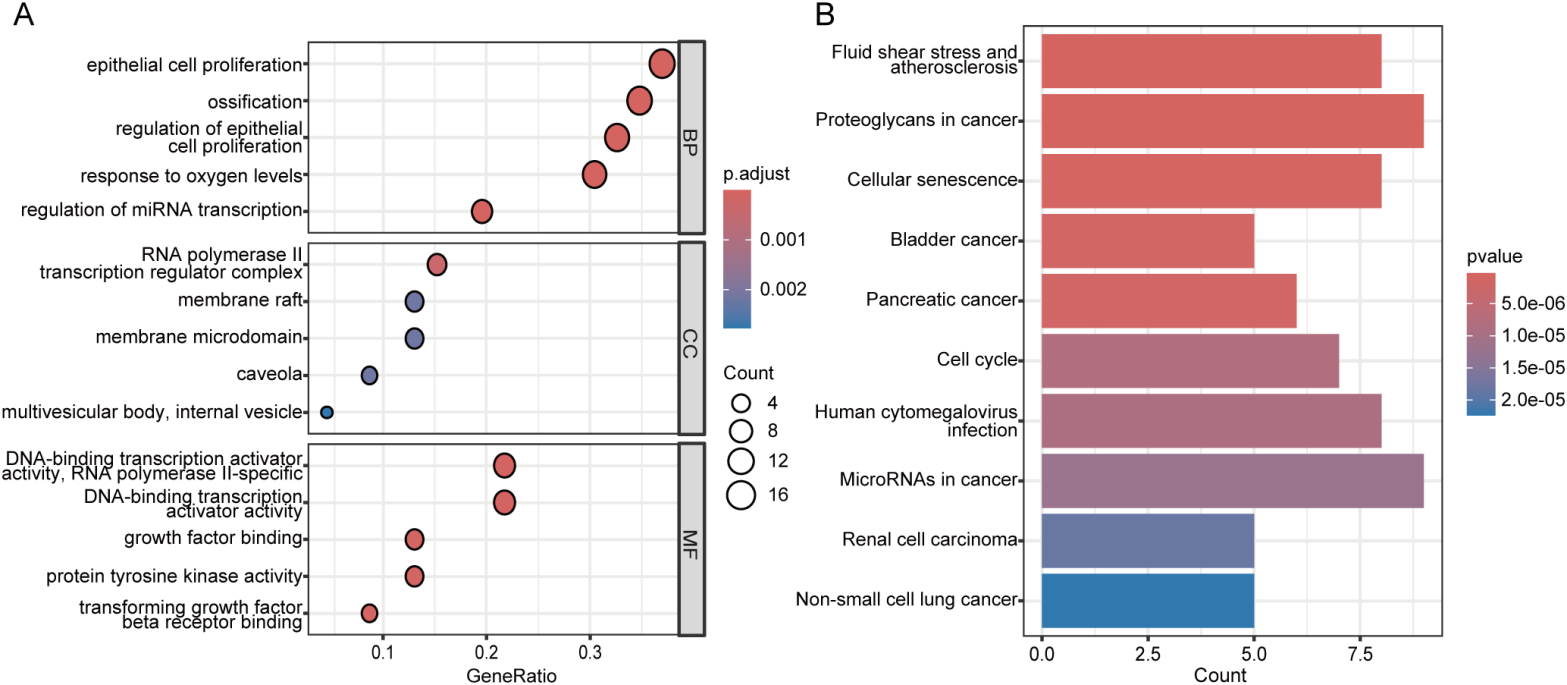
Functional enrichment analysis of genes in the MCODE clusters. **(A)** Bubble plot of Gene Ontology (GO) enrichment analysis for genes in the eight MCODE clusters. The bubble size indicates the number of genes in each GO term, and the color represents the adjusted p-value. **(B)** Bar plot of KEGG pathway enrichment analysis for genes in the eight MCODE clusters. The bar length indicates the number of genes in each pathway, and the bar color reflects the p-value. BP, Biological Process; MF, molecular function; CC, cellular component.

Subsequently, we performed GO and KEGG enrichment analysis on these 46 genes. The GO analysis revealed enrichment in terms such as epithelial cell proliferation, ossification, RNA polymerase II transcription regulator complex, caveola, and DNA-binding transcription activator activity (**Figure 5A**). The KEGG analysis highlighted pathways including proteoglycans in cancer, microRNAs in cancer, fluid shear stress and atherosclerosis, cellular senescence, and human cytomegalovirus infection (**Figure 5B**). These findings underscore the potential roles of the identified modules in the molecular mechanisms underlying IPF.

### 3.4 Identification and functional analysis of core CS-DEGs

**Figure 6:**
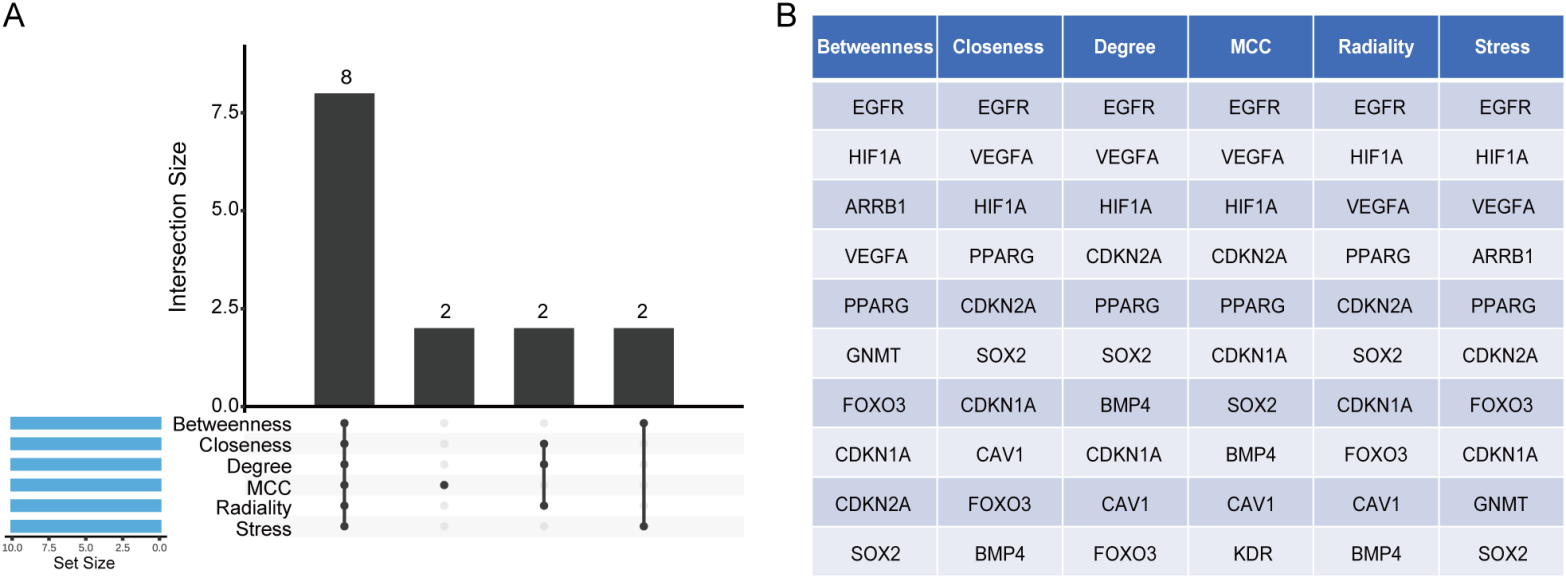
Identification of core CS-DEGs. **(A)** Venn diagram showing the eight overlapping core CS-DEGs identified through six different algorithms. **(B)** Table displaying the top ten genes identified by each algorithm used for core CS-DEG selection.

To identify the core CS-DEGs, we analyzed the 106 genes from the PPI network using the Cytohubba plugin in Cytoscape. We employed six algorithms—Betweenness, Closeness, Degree, MCC, Radiality, and Stress—to determine the top ten scoring genes. A Venn analysis of these results revealed eight overlapping core genes: VEGFA, HIF1A, EGFR, PPARG, SOX2, CDKN2A, FOXO3, and CDKN1A (**Figures 6A, B**).

**Figure 7:**
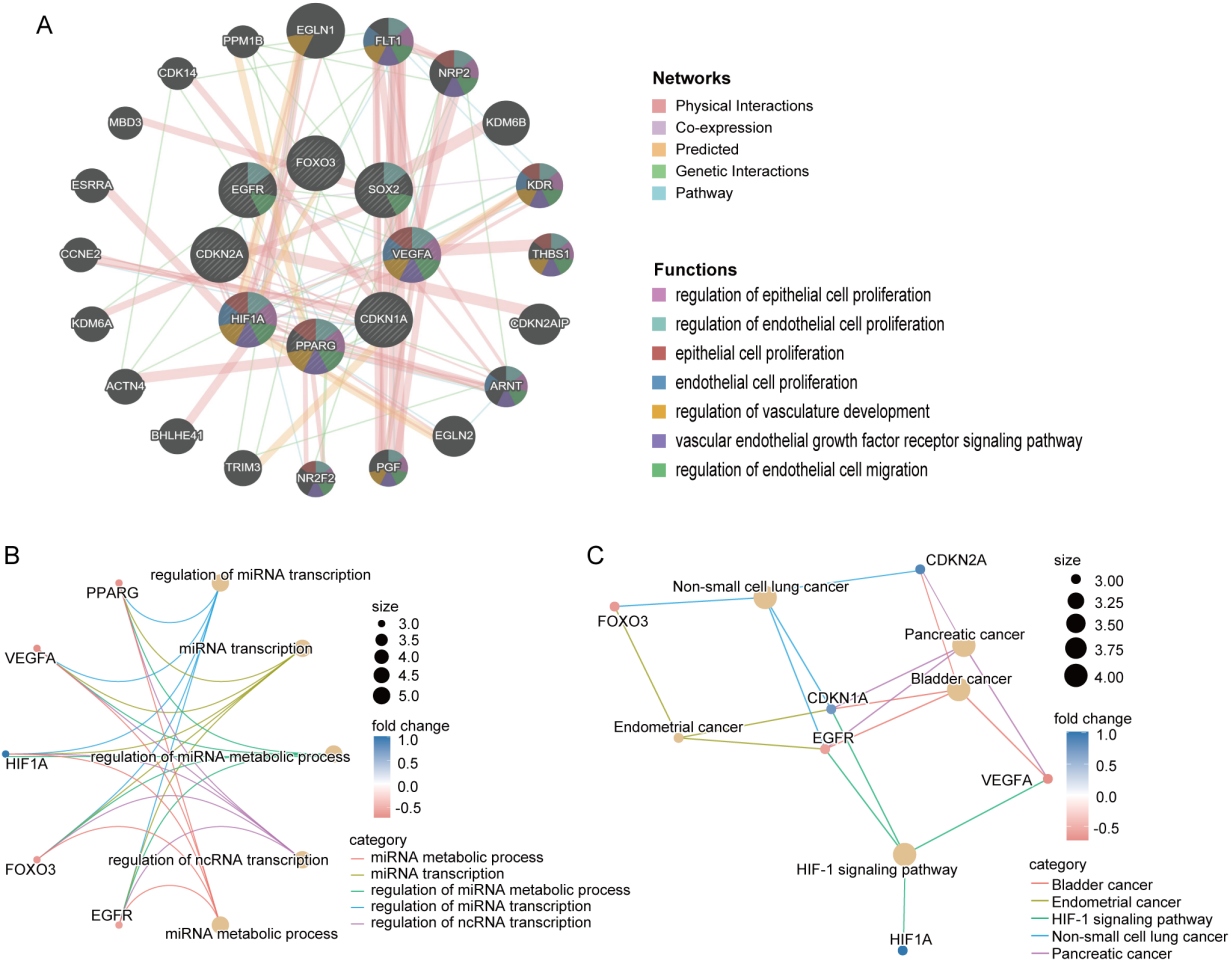
Interaction and functional enrichment analysis of core CS-DEGs. **(A)** Gene-genes interaction network of core CS-DEGs analyzed using the GeneMANIA database. Nodes represent the 20 most significantly altered neighboring genes, with edge color indicating the type of interaction and node color representing gene function. Network plot of GO analysis **(B)** and KEGG pathway enrichment analysis **(C)** for core CS-DEGs.

We then conducted a functional analysis of these eight core CS-DEGs. Utilizing the GeneMANIA database, we constructed a co-expression network that showed 61.91% of interactions were physical, 16.37% were based on co-expression, 13.90% were predicted interactions, 6.73% were genetic interactions, and 1.09% were pathway related. These core genes were primarily associated with functions such as the regulation of epithelial cell proliferation, regulation of endothelial cell proliferation, epithelial cell proliferation, endothelial cell proliferation, and regulation of vasculature development (**Figure 7A**).

Additionally, the GO analysis indicated that these genes are linked to processes including miRNA metabolic process, miRNA transcription, regulation of miRNA metabolic process, regulation of miRNA transcription, and regulation of ncRNA transcription (**Figure 7B**). The KEGG pathway analysis demonstrated significant enrichment in pathways related to bladder cancer, endometrial cancer, the HIF-1 signaling pathway, non-small cell lung cancer, and pancreatic cancer (**Figure 7C**).

### 3.5 Immune cell infiltration analysis and correlation with core CS-DEGs

**Figure 8:**
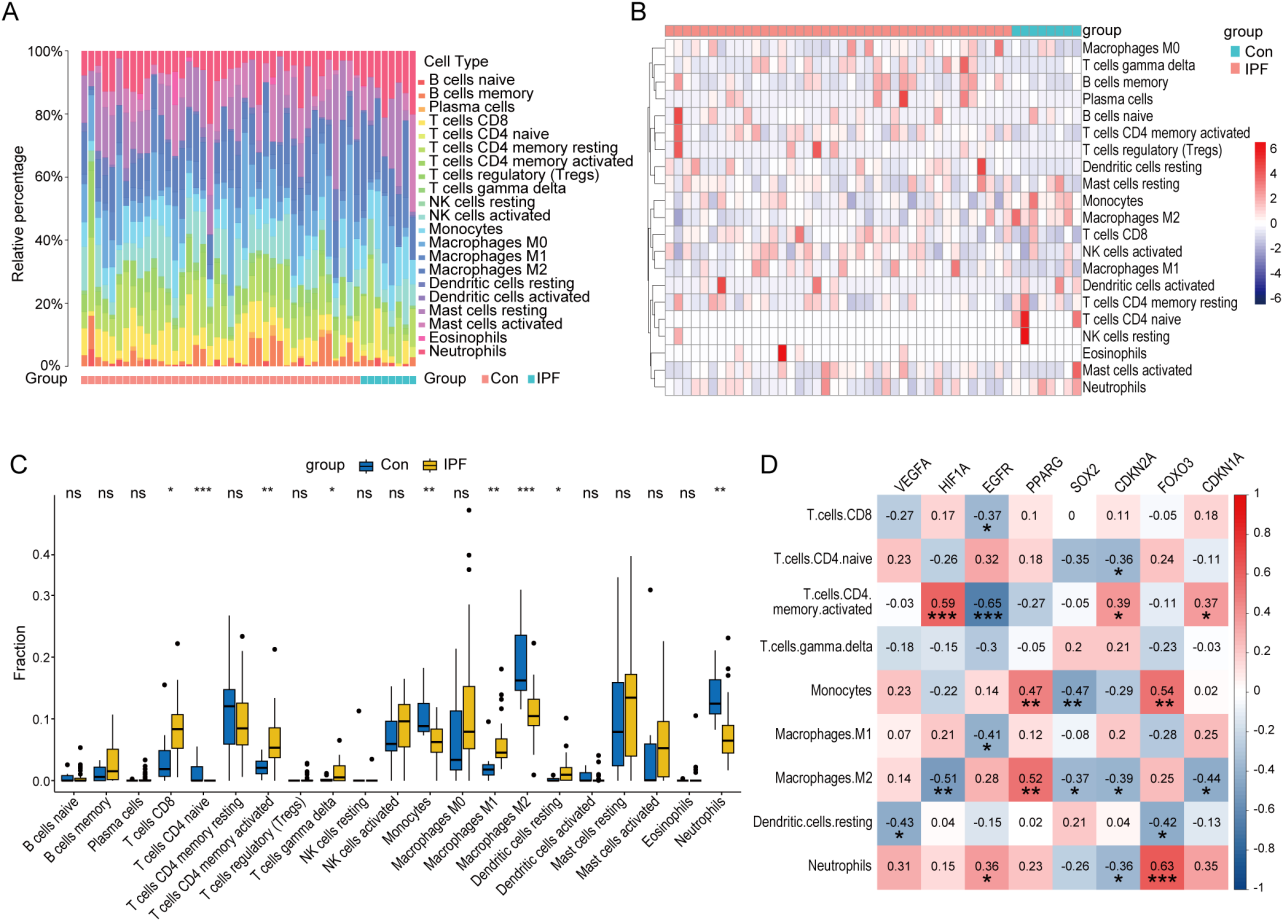
Immune characteristic analysis in IPF and healthy control lung tissues. Histogram **(A)**, heatmap **(B)**, and boxplot **(C)** comparing the infiltration ratios of 22 immune cell types between the IPF group and the healthy control group. **(D)** Correlation plot between core CS-DEGs and immune cells with differential infiltration. The numbers in the squares represent the correlation coefficients, with red indicating positive correlation and blue indicating negative correlation. ns P ≥ 0.05; *P < 0.05; **P < 0.01; ***P < 0.001.

Previous functional analyses of DEGs and core CS-DEGs indicated a close association with immune processes. To further investigate changes in the immune microenvironment in IPF, we employed the CIBERSORT algorithm to analyze the infiltration of 22 immune cell types (**Figures 8A, B**), comparing the immune cell profiles between the IPF group and healthy controls. The results showed that in IPF lung tissue, the proportions of T cells CD8, T cells CD4 memory activated, T cells gamma delta, Macrophages M1, and Dendritic cells resting were significantly elevated compared to healthy controls. Conversely, the proportions of T cells CD4 naive, Monocytes, Macrophages M2, and Neutrophils were significantly decreased (**Figure 8C**).

We then performed correlation analysis between these nine immune cell types and the eight core CS-DEGs. The results indicated that HIF1A was positively correlated with T cells CD4 memory activated but negatively correlated with Macrophages M2. EGFR negatively correlated with T cells CD4 memory activated, while PPARG positively correlated with Macrophages M2. Additionally, FOXO3 was positively correlated with both Monocytes and Neutrophils (**Figure 8D**).

### 3.6 Identification of key CS-DEGs with diagnostic value in IPF

**Figure 9:**
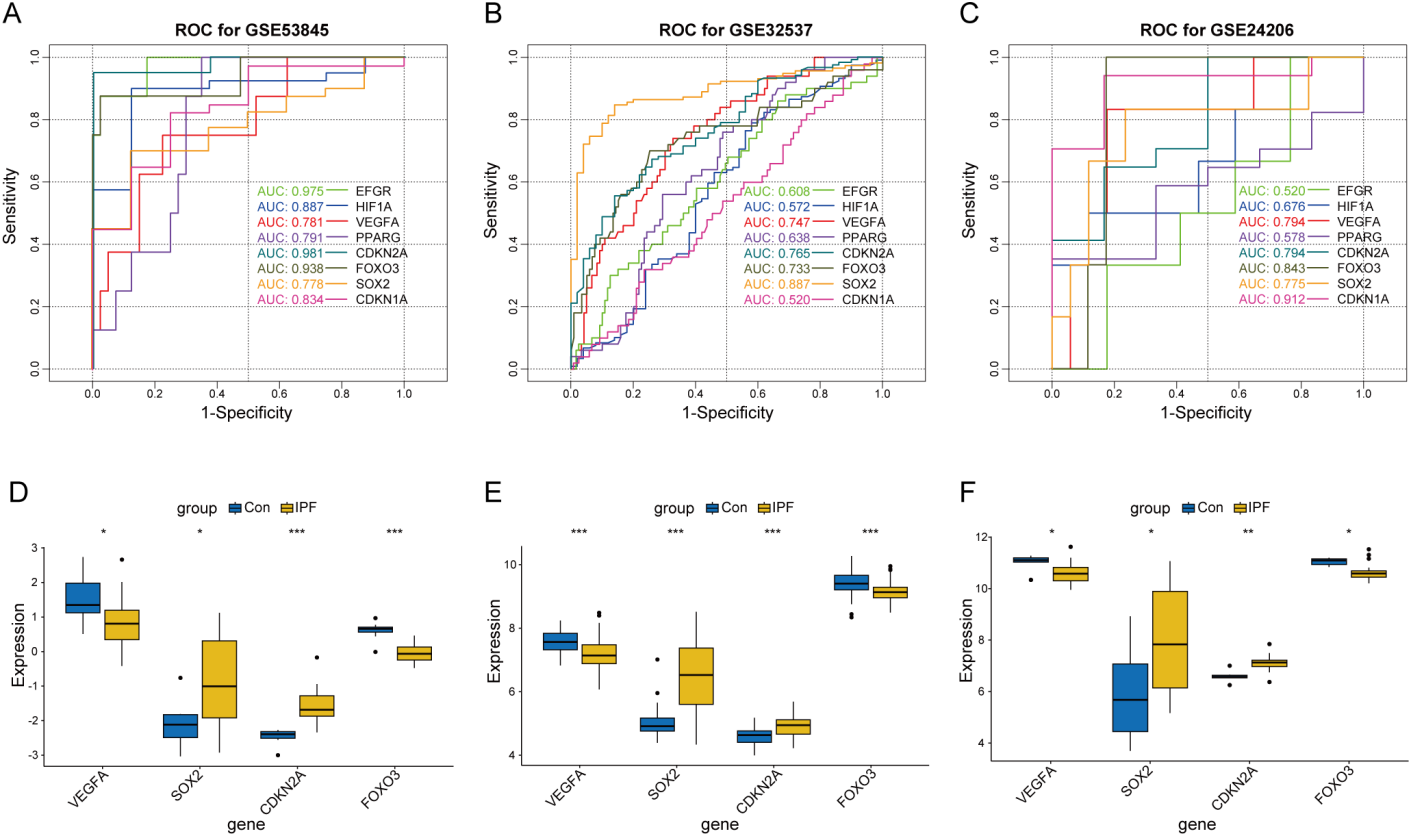
Screening of key CS-DEGs based on the diagnostic value of core CS-DEGs in IPF. ROC curves for core CS-DEGs in datasets GSE53845 **(A)**, GSE32537 **(B)**, and GSE24206 **(C)**, respectively. The curves represent the performance of different genes, with curve colors corresponding to each gene. Boxplots showing the expression levels of key CS-DEGs in the IPF group and healthy control group for datasets GSE53845 **(D)**, GSE32537 **(E)**, and GSE24206 **(F)**. ns P ≥ 0.05; *P < 0.05; **P < 0.01; ***P < 0.001.

To assess the diagnostic potential of the eight core CS-DEGs in IPF, we performed ROC analysis across the GSE53845, GSE32537, and GSE24206 datasets, using an AUC > 0.7 as the criterion for diagnostic relevance. This approach identified four key CS-DEGs—VEGFA, SOX2, CDKN2A, and FOXO3—with significant diagnostic value (**Figures 9A–C**). VEGFA and FOXO3 had reduced expression in IPF lung tissue, while SOX2 and CDKN2A were upregulated (**Figures 9D–F**).

**Figure 10:**
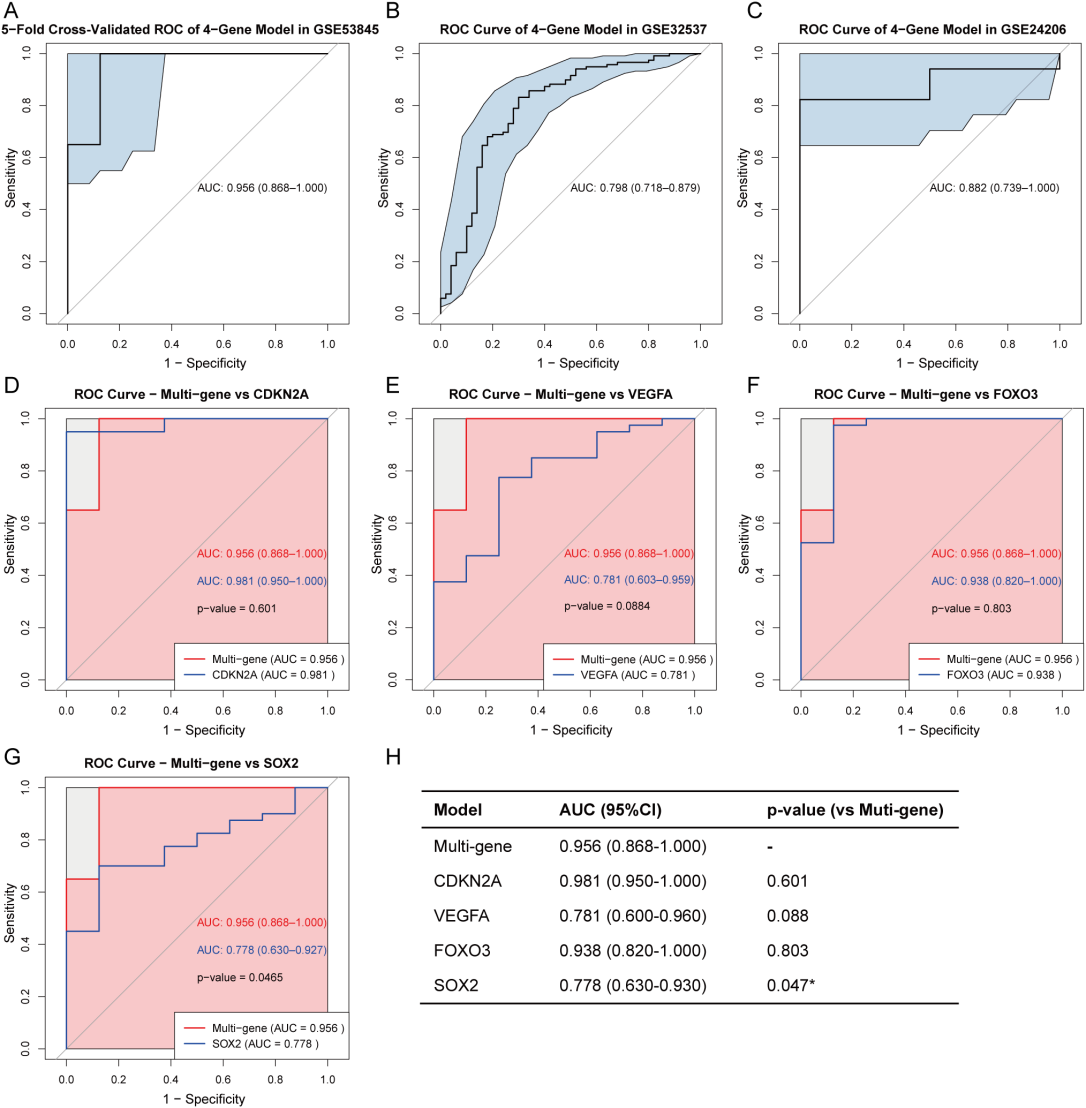
Diagnostic performance of the 4-gene model in IPF. Five-fold cross-validated ROC curve of the LASSO logistic regression model using four key CS-DEGs (CDKN2A, VEGFA, FOXO3, and SOX2) in the training set (GSE53845) **(A)**. External validation of the multi-gene model in two independent validation sets: GSE32537 **(B)** and GSE24206 **(C)**. AUC values with 95% confidence intervals (CI) are shown. Comparison of ROC curves between the multi-gene model and each individual gene: CDKN2A **(D)**, VEGFA **(E)**, FOXO3 **(F)**, and SOX2 **(G)** in the training set. P-values are calculated using DeLong’s test. **(H)** Summary table of AUC values (95% CI) for the multi-gene and single-gene models, and their statistical comparison with the multi-gene model using DeLong’s test. *P < 0.05.

To evaluate the diagnostic performance of the 4-gene signature (CDKN2A, VEGFA, FOXO3, and SOX2) in IPF, we first constructed a LASSO logistic regression model using the GSE53845. The 5-fold cross-validated ROC curve demonstrated a high AUC of 0.956 (95% CI: 0.868-1.000), indicating excellent discriminatory ability (**Figure 10A**). The model’s robustness was confirmed in two independent datasets: GSE32537 (AUC = 0.798, 95% CI: 0.718-0.879) and GSE24206 (AUC = 0.882, 95% CI: 0.739-1.000) (**Figures 10B, C**). We next compared the 4-gene model with each key CS-DEG in the GSE53845. The 4-gene model showed higher AUC than any single marker—CDKN2A (0.981), VEGFA (0.781), FOXO3 (0.938), and SOX2 (0.778) (**Figures 10D–G**). DeLong’s test indicated a significant improvement over SOX2 (p = 0.047), whereas differences versus the other genes were not statistically significant (**Figure 10H**). Overall, integration of four key CS-DEGs improves diagnostic accuracy, underscoring the clinical potential of this 4-gene model for IPF.

**Figure 11:**
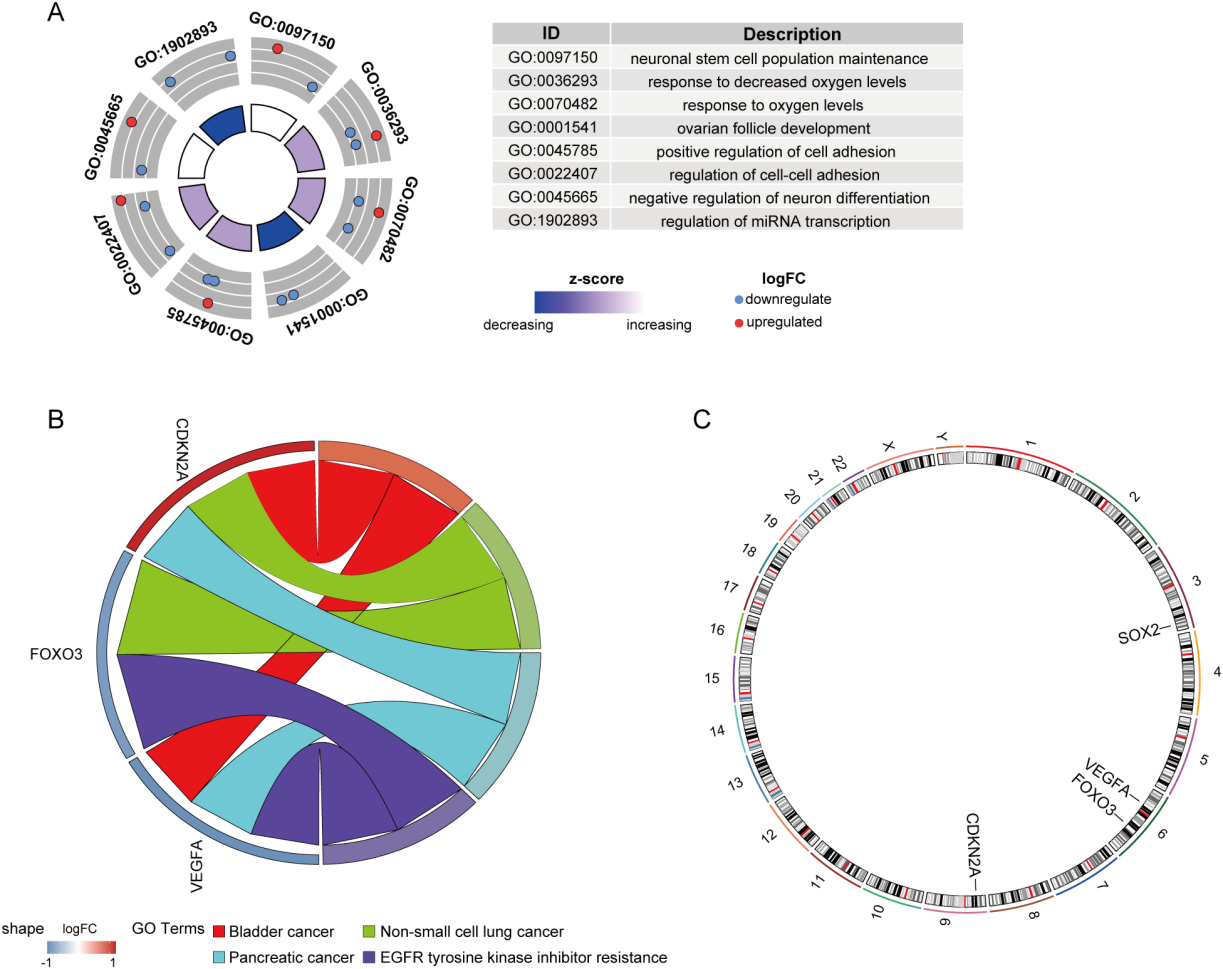
Functional enrichment and chromosomal localization of key CS-DEGs. **(A)** GO analysis results for key CS-DEGs. Node colors represent gene expression levels, and the color of the rectangles indicates the Z-score. **(B)** Chord diagram showing the KEGG pathway enrichment analysis for key CS-DEGs. **(C)** Chromosomal localizations of four key CS-DEGs.

Further functional analysis revealed that these genes are involved in neuronal stem cell population maintenance, response to decreased oxygen levels, response to oxygen levels, ovarian follicle development, and positive regulation of cell adhesion(GO) (**Figure 11A**), and are enriched in pathways related to Bladder cancer, Non-small cell lung cancer, Pancreatic cancer, and EGFR tyrosine kinase inhibitor resistance(KEGG) (**Figure 11B**). **Figure 11C** displays the chromosomal locations of these key genes.

### 3.7 Construction of TF, miRNA, and drug networks targeting key CS-DEGs in IPF

**Figure 12:**
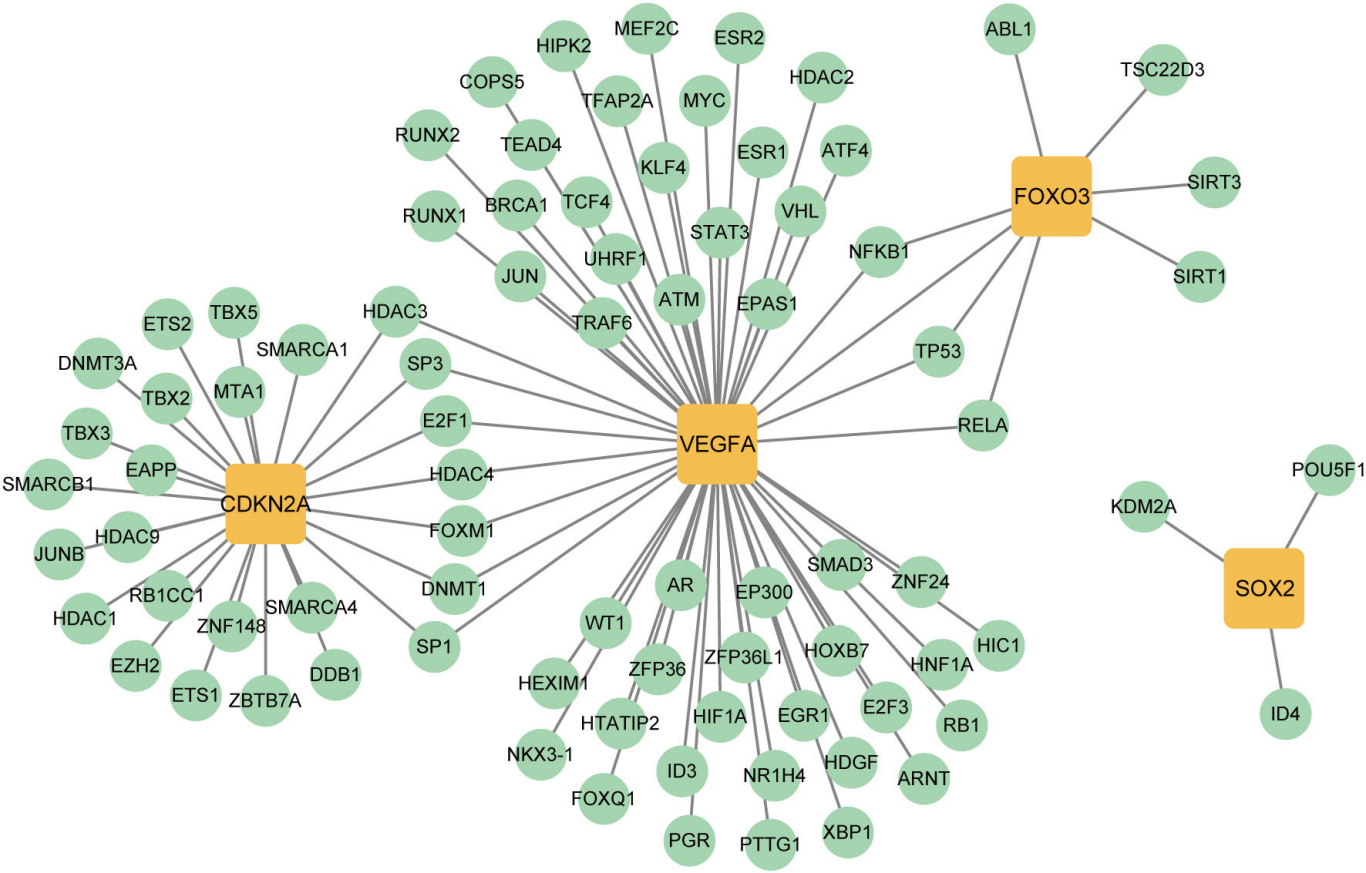
Transcription factors (TFs)- key CS-DEGs interaction network. The green circles represent TFs, and the yellow squares represent key CS-DEGs.

**Figure 13:**
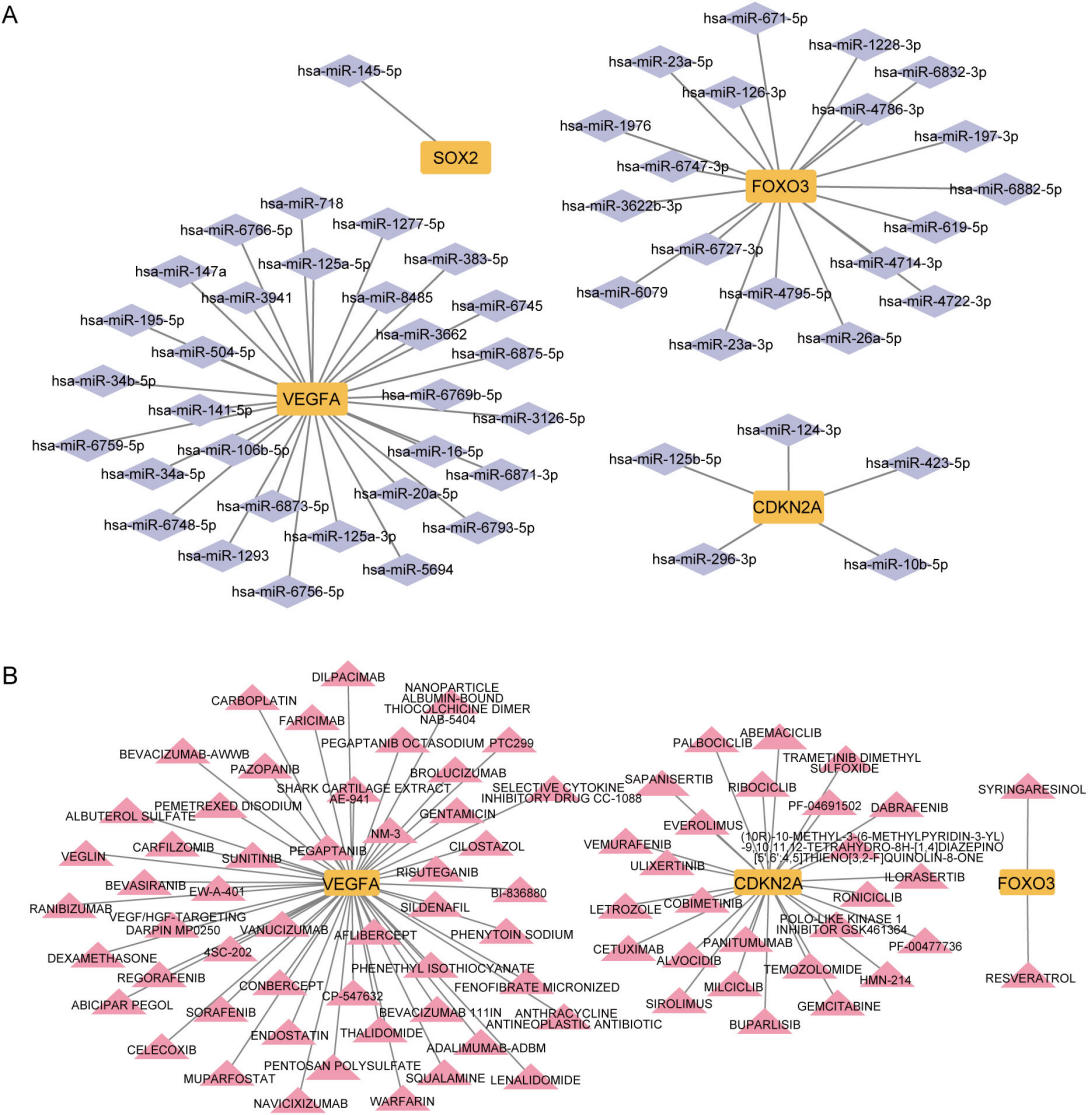
Micro RNAs (miRNAs) and drugs regulation of key CS-DEGs. **(A)** miRNAs - key CS-DEGs interaction network. The purple diamonds represent miRNAs, and the yellow squares represent key CS-DEGs. **(B)** Drugs - key CS-DEGs interaction network. The pink triangles represent drugs, and the yellow squares represent key CS-DEGs.

To explore transcriptional regulation for the four key CS-DEGs, we constructed separate regulatory networks of transcription factors (TFs) (**Figure 12**) and miRNAs (**Figure 13A**). The analysis revealed seven TFs (HDAC3, SP3, E2F1, HDAC4, FOXM1, DNMT1, SP1) regulating both CDKN2A and VEGFA, and three TFs (NFKB1, TP53, RELA) influencing both VEGFA and FOXO3. Notably, FOXO3, one of the key CS-DEGs, also acts as a TF regulating VEGFA expression. The analysis identified 1, 5, 19, and 30 miRNAs targeting SOX2, CDKN2A, FOXO3, and VEGFA, respectively.

To identify drugs with potential therapeutic effects on these key CS-DEGs, we constructed a drug-gene interaction network (**Figure 13B**). This analysis revealed 2, 26, and 52 candidate drugs with potential effects on FOXO3, CDKN2A, and VEGFA, respectively.

### 3.8 Validation of key CS-DEGs in IPF lung tissue

**Figure 14:**
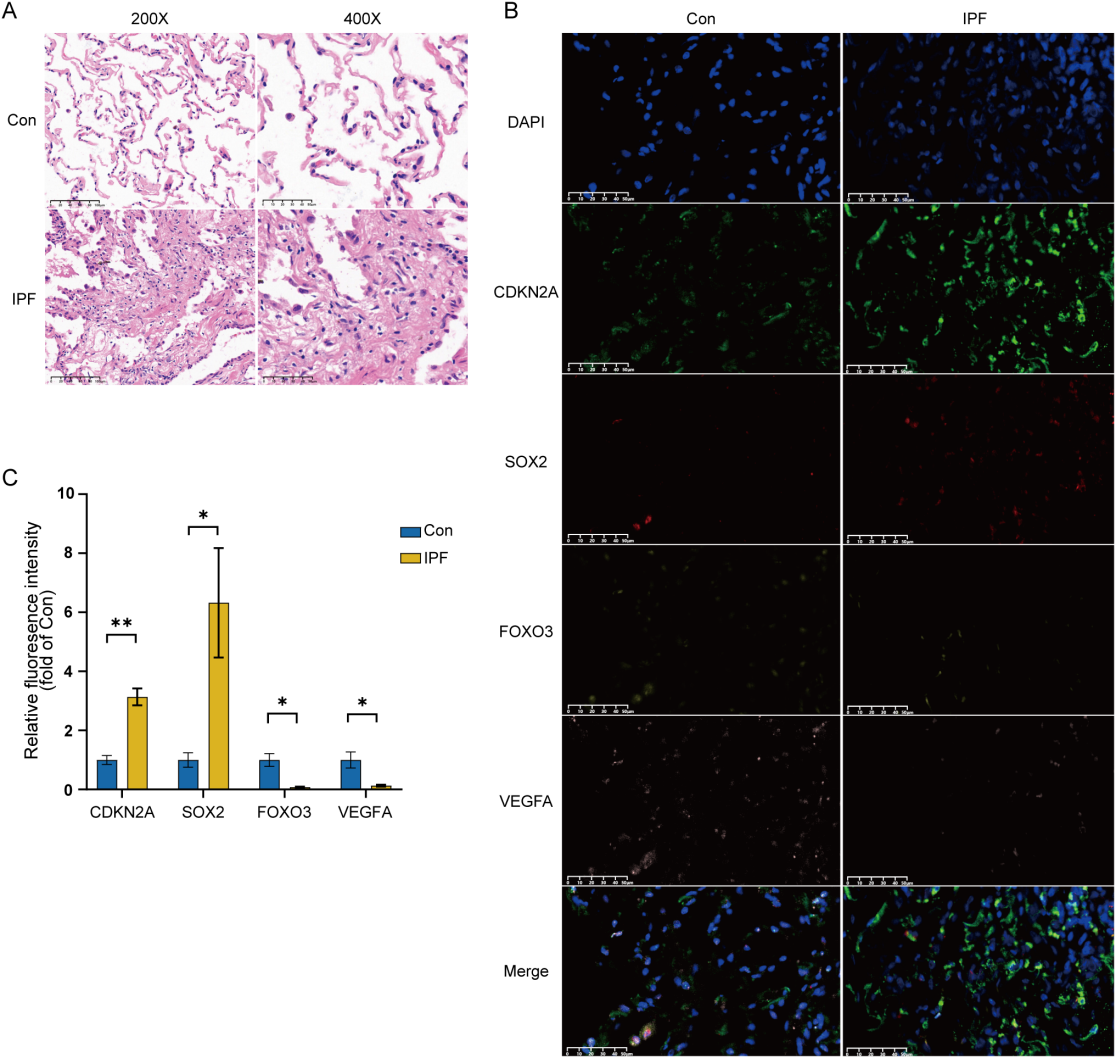
Experimental validation of key CS-DEGs in clinical samples. **(A)** H&E staining of IPF lung tissue and normal control lung tissue, showing images at 200× and 400× magnification. **(B)** Representative immunofluorescence images of CDKN2A (green), SOX2 (red), FOXO3 (yellow), and VEGFA (pink) expression in IPF lung tissue and normal control lung tissue (magnification: 400×). DAPI, 4′,6-diamidino-2-phenylindole. **(C)** Quantitative histogram of relative fluorescence intensity (n=3), with the y-axis representing fluorescence intensity relative to the healthy control group. *P < 0.05; **P < 0.01.

To further validate the four key CS-DEGs, we performed H&E staining and immunofluorescence staining on clinical samples. H&E staining revealed significant fibrotic changes in IPF lung tissue, including disrupted alveolar structure, thickened alveolar walls, inflammatory cell infiltration, and dense collagen deposition (**Figure 14A**).

Immunofluorescence staining showed that the average fluorescence intensity of CDKN2A and SOX2 was significantly higher in IPF lung tissue compared to healthy controls, while FOXO3 and VEGFA expression were significantly lower in IPF samples (**Figure 14B**). Quantitative analysis confirmed these differential expression patterns, validating the bioinformatics results and highlighting the importance of CDKN2A, VEGFA, SOX2, and FOXO3 in IPF pathogenesis (**Figure 14C**).

## 4 Discussion

IPF is a fatal, age-related interstitial lung disease characterized by fibroblast activation and ECM deposition (1). Emerging evidence suggests that cellular senescence in various cell types, including alveolar epithelial cells, fibroblast, and mesenchymal stem cell, plays a crucial role in the initiation of IPF (5, 18, 19). Cellular senescence contributes to the disease through two primary mechanisms: impaired regeneration of alveolar epithelial cells and the compromised recovery of alveolar structure and function create a pro-fibrotic environment (1, 19–21); additionally, the paracrine effects of the SASP promote the accumulation of senescent cells in the pulmonary microenvironment, exacerbating tissue damage and fibrosis (13, 22). Given the involvement of cellular senescence in IPF and other age-related diseases, targeting senescent cells or blocking SASP has emerged as a promising therapeutic strategy (7, 11, 12).

In this study, we aimed to explore the molecular mechanisms underlying cellular senescence in IPF. We identified 122 CS-DEGs, including 60 upregulated and 62 downregulated genes. Through a multi-step bioinformatics approach, four key CS-DEGs—CDKN2A, VEGFA, SOX2, and FOXO3—were selected based on their consistent diagnostic performance across datasets (AUC > 0.7). Their differential expression in IPF lung tissues was subsequently validated by multiplex immunofluorescence, confirming their potential as diagnostic biomarkers. Moreover, a multi-gene logistic regression model incorporating these four genes demonstrated excellent overall predictive performance (AUC = 0.956). Although the single-gene model based on CDKN2A showed an even higher AUC (0.981), the difference did not reach statistical significance, potentially indicating the pivotal role of CDKN2A in senescence-associated pathways. In contrast, SOX2 exhibited relatively poor diagnostic power as a single marker (AUC = 0.778), with significantly lower performance compared to the multi-gene model, suggesting its limited utility as a standalone biomarker. These findings imply that a composite multi-gene model is more suitable for capturing disease heterogeneity and enhancing diagnostic accuracy in IPF. Nonetheless, further clinical studies are required to validate its applicability in real-world settings.

CDKN2A is a cyclin-dependent kinase inhibitor gene that encodes p16INK4a, a critical regulator of cell cycle progression (7, 23, 24). By inhibiting cyclin-dependent kinase 4/6 (CDK4/6), p16INK4a reduces phosphorylation of retinoblastoma protein (pRB), thereby suppressing the activation of the downstream transcription factor E2F and leading to irreversible cell cycle arrest (5, 25). This regulatory axis acts independently or synergistically with the p53–p21 pathway, forming two central branches of the cellular senescence program (11, 25). In our study, CDKN2A was significantly upregulated in IPF lung tissues, and KEGG enrichment analysis revealed its strong association with cellular senescence and p53 signaling pathway. Mechanistically, this is consistent with the persistent injury and oxidative stress present in IPF lungs, which induce CDKN2A expression, activate the p16 and p53 pathways, and drive alveolar epithelial cell cycle arrest and the establishment of a SASP (26). This senescent phenotype, in turn, promotes fibroblast activation and ECM deposition—key pathological hallmarks of IPF. Our findings are corroborated by prior studies. Lee et al. reported increased CDKN2A expression in epithelial and fibroblast populations of IPF lungs using single-cell RNA sequencing, while Xu et al. observed that elevated CDKN2A expression was associated with decreased pulmonary function in IPF patients (12, 23, 27). In preclinical models, inhibition of CDKN2A or selective clearance of p16^+^ fibroblasts—such as with the senolytic compound XL888—has been shown to alleviate fibrosis (23, 27). Collectively, these results provide mechanistic evidence linking CDKN2A-driven senescence pathways to the pathogenesis of IPF, and support the potential of CDKN2A as both a diagnostic biomarker and a therapeutic target.

VEGFA, a 34–46 kDa glycoprotein, functions as a pro-inflammatory cytokine and a key regulator of angiogenesis, playing multifaceted roles in lung injury and fibrosis (28, 29). In this study, we observed a significant downregulation of total VEGFA in IPF lung tissues compared to healthy controls. However, VEGFA levels in the bronchoalveolar lavage fluid (BALF) of IPF patients remain controversial, with reports of either decreased or unchanged expression (30–32). This discrepancy may reflect spatial heterogeneity: senescence of parenchymal endothelial cells may drive global depletion of VEGFA within the lung tissue, while transient secretion from alveolar macrophages or activated fibroblasts in early fibrotic niches may sustain localized VEGFA pools. The dual role of VEGFA—pro-fibrotic effects (e.g., promoting fibroblast proliferation and migration) versus protective functions (e.g., enhancing alveolar repair and activating NK cell–mediated immune responses)—further complicates its therapeutic targeting (30, 33, 34). Emerging evidence suggests that this dichotomy is largely driven by isoform-specific effects. Alternative splicing generates functionally distinct isoforms such as the pro-angiogenic VEGF-A165a and the anti-angiogenic VEGF-A165b, which exert differential effects on fibroblast activity and ECM production. The spatiotemporal balance between these isoforms may critically influence disease progression (28). In our study, VEGFA was identified as a high-confidence biomarker. However, bulk RNA-seq and immunofluorescence are limited in their ability to distinguish among isoforms. Future studies should integrate single-cell transcriptomics (to map isoform-specific cellular origins) with spatially resolved proteomics (to quantify VEGF-A165a/b ratios within fibrotic niches) to reconcile these molecular layers. Clinically, isoform-selective interventions (e.g., neutralization of VEGF-A165a while preserving VEGF-A165b) or compartment-targeted delivery strategies (e.g., inhalable biologics) may offer precise therapeutic approaches by balancing the opposing actions of VEGFA in IPF.

SOX2 is a transcription factor essential for maintaining embryonic stem cells and inducing pluripotency, and plays a pivotal role in airway epithelial homeostasis and repair (35). In our study, SOX2 was significantly upregulated in IPF lung tissues compared to healthy controls, consistent with previous findings (36). GO enrichment analysis revealed that SOX2-related genes were enriched in biological processes such as neuronal stem cell population maintenance, response to oxygen levels, and positive regulation of cell adhesion. KEGG enrichment further emphasized its involvement in epithelial and endothelial cell proliferation and its regulation. These results suggest that SOX2 may participate in modulating epithelial progenitor cell fate and aberrant repair responses within the fibrotic lung microenvironment. Under conditions of hypoxia and injury-induced stress, the upregulation of SOX2 in IPF lungs may drive compensatory epithelial proliferation. However, excessive or dysregulated SOX2 activity may also promote airway-like differentiation of alveolar epithelial cells, contributing to distal airspace bronchiolization—a histopathological hallmark of IPF (37, 38). In addition, SOX2 has been shown to promote the expression of fibroblast growth factor 4 (FGF4) or interacting with Smad3 to influence cell proliferation (36). These mechanisms may further facilitate fibroblast activation and ECM remodeling. Recent studies have identified pentraxin 3 (PTX3) as a potential SOX2 inhibitor, capable of counteracting abnormal epithelial remodeling and alveolar destruction while preventing fibroblast-associated stemness and collagen synthesis (39). These findings suggest that targeting SOX2 and its regulatory pathways could offer novel therapeutic strategies for IPF.

FOXO3, a member of the FOXOs transcription factor family, is characterized by its conserved forkhead box (FOX) DNA-binding domain and plays a critical role in biological processes such as proliferation, apoptosis, and differentiation (40, 41). In our study, FOXO3 expression was significantly downregulated in IPF lung tissues compared to healthy controls, which aligns with previous reports (40, 41). Accumulating evidence suggests that FOXO3 acts as a critical suppressor in IPF fibrogenesis (40–42). By inhibiting FOXO3 activity, IPF lung fibroblasts maintain their pathological phenotype, characterized by enhanced proliferation, resistance to apoptosis, and excessive collagen matrix production, thereby promoting disease progression. Interestingly, our study further revealed that FOXO3 expression positively correlates with neutrophil and monocyte infiltration in IPF lung tissues. It has been well-documented that immune responses play a pivotal role in IPF development, where dysregulated interactions between epithelial cells and immune cells can trigger EMT and sustain chronic inflammation, thus accelerating fibrosis (33, 43). FOXO3 has been identified as a key nuclear factor regulating EMT in IPF (44). Given its multifaceted role, therapeutic reactivation of FOXO3 has emerged as a promising strategy for IPF treatment. Hamza et al. identified UCN-01, a staurosporine derivative, as a potential agent capable of reactivating FOXO3, reversing fibroblast phenotypic changes, and ameliorating pulmonary fibrosis (40).

Our study has several limitations. First, while the integration of three independent datasets strengthens the reliability of our findings through cross-validation, the sample size of each individual cohort remains modest. This may limit the detection of genes with subtle expression changes or low abundance. Future studies with larger cohorts are needed to confirm these results. Second, the publicly available datasets lack detailed clinical information such as smoking history, disease severity stages, and longitudinal pulmonary function data. These missing variables could act as potential confounders, and their impact should be addressed in future prospective studies. Third, while our immunofluorescence analysis confirmed the dysregulation of CDKN2A, SOX2, FOXO3, and VEGFA at the protein level, further functional studies are required to establish their mechanistic roles in IPF. As an initial bioinformatics-driven investigation, this study focused on identifying and validating robust CS-DEG signatures with diagnostic potential. The consistent identification of these genes across multiple datasets highlights their promise as key contributors to senescence and fibrosis, inviting deeper experimental exploration in future work.

## 5 Conclusion

In conclusion, this study underscores the pivotal role of cellular senescence in the pathogenesis of idiopathic pulmonary fibrosis (IPF) and identifies four key CS-DEGs (CDKN2A, VEGFA, SOX2, and FOXO3) as potential biomarkers for diagnosis and targets for therapy. Our findings provide a foundation for future research focused on developing senescence-based interventions, which could improve clinical outcomes for IPF patients.

## Data Availability

The original contributions presented in the study are included in the article/supplementary material. Further inquiries can be directed to the corresponding author.
